# Editorial: Synergistic interactions between exosomes and tunneling nanotubes in long-range intercellular transfer

**DOI:** 10.3389/fnmol.2023.1240959

**Published:** 2023-07-31

**Authors:** Sangeeta Nath, Dean L. Pountney, Jiri Neuzil

**Affiliations:** ^1^Manipal Institute of Regenerative Medicine, Bangalore, India; ^2^Manipal Academy of Higher Education, Manipal, India; ^3^School of Pharmacy and Medical Science, Griffith University, Southport, QLD, Australia; ^4^Institute of Biotechnology, Czech Academy of Sciences, Prague-West, Czechia

**Keywords:** exosomes, tunneling nanotubes, intercellular transfer, neurodegenerative aggregates, cell-to-cell communication

Cell-to-cell communication is a critical process that ensures interaction between adjacent and distant cells to maintain cellular homeostasis in normal physiology and pathophysiological conditions. Two adjacent cells communicate to transfer ions and small molecules via Gap junctions, small selective ion channels. Distant cells establish long-range communication via endocrine/paracrine secretions, extracellular vesicles (EVs) and tunneling nanotubes (TNTs) (Aguzzi and Lakkaraju, 2016). Recent studies have implicated crucial roles of exosomes and TNTs in long-range intercellular transfer of cellular materials. The exosome is the smallest of the EVs, moving between physically distant cells to exchange cellular materials, including mRNA and biomolecules. Emerging studies have shown transfer of mitochondria via exosomes between distant cells (Hough et al., 2018). In parallel, TNTs make direct actin-membrane open-ended channels to transfer cytoplasmic materials such as mRNA, proteins, and various organelles between distal cells. Recent advancements in the understanding of long-range intercellular transfer by exosomes and TNTs have demonstrated profound implications in various (patho)physiological conditions, including cancer progression, immune responses, neurodegeneration and tissue repair (Raghavan et al., 2021).

Emerging evidence suggests that cellular stress can trigger the biogenesis of these two intercellular transfer modes, i.e., both exosomes and TNTs. Studies have demonstrated that neurodegenerative protein aggregates facilitate exosome release and biogenesis of TNTs to enhance transfer of toxic materials (Victoria and Zurzolo, 2017; Rastogi et al., 2021). Other recent studies have indicated the therapeutic potential of exosomes and TNTs to counteract the detrimental effects of toxicities. Exosomes derived from neuronal cells may contain neuroprotective factors and transfer between cells to promote neuronal survival (Natale et al., 2022). Furthermore, exosomes derived from mesenchymal stem cells (MSCs) show great potential to promote tissue regeneration and repair by stimulating cell proliferation and angiogenesis (Raghavan et al., 2021). Similarly, transfer of healthy mitochondria from MSCs via TNTs demonstrated their crucial role in cell survival (Spees et al., 2006). TNT-mediated intercellular transfer of mitochondria from MSCs can rescue retinal ganglion cells and corneal epithelial in dry eye disease (Jiang et al., 2023). Similarly, exosomes released from Muller glia are potentially neurotrophic and may have therapeutic applications, Kalargyrou et al..

Interestingly, studies have implicated two disparate TNT-based fates due to cell-to-cell transfer of aggregation-prone proteins, the spreading of pathology and rescuing of toxic burdens of aggregates. The review by Lagalwar, 2022 discusses these two disparate TNT-based fates in neurodegenerative pathologies, such as Alzheimer's, Parkinson's, and Huntington's disease, and ALS. After the discovery of the TNT-structure (Rustom et al., 2004), several studies have shown TNT-based spreading of aggregation-prone proteins and pathology progression in neuronal models (Victoria and Zurzolo, 2017). On the other hand, several studies have shown that astrocytes and microglia rescue toxicities of neurodegenerative aggregates by sharing their burden with neighboring cells by means of TNTs (Rostami et al., 2017; Scheiblich et al., 2021). Moreover, one study demonstrated that neuronal cells transfer toxic materials to microglia, whereas microglia can transfer healthy mitochondria to rescue neurons and to ameliorate neurodegeneration (Chakraborty et al., 2023). This implicates the intricate interplay between intercellular transfer and cellular stress responses.

Cellular stress induced triggering of molecular events, which are associated with endolysosomal toxicities, mitochondrial toxicities, oxidative stress and intracellular trafficking interface to the biogenesis of both exosomes and TNTs (Nawaz and Fatima, 2017). This raises the question whether cells use these modes of intercellular transfer to rescue cells by facilitating clearance of toxic burdens. Moreover, cells possess other intriguing mechanisms to clear or degrade unwanted material. In addition to the conventional lysosomal and proteasomal degradation machineries, cells can activate autophagosome dependent cellular clearance in response to pathological toxic conditions. Enhanced upregulation of autophagy is evident in all neurodegenerative diseases. The perspective of intercellular transfer on leaderless neurodegenerative proteins in relation to autophagosome dependent cellular clearance has been discussed in Padmanabhan and Manjithaya. Further, studies are needed to understand the fate of conventional and autophagosome-mediated cellular clearance involving exosomes and TNT-mediated rescue of toxicities. Nevertheless, several questions remain unanswered. What factors trigger these intercellular transfer vehicles? Do dysfunction or inefficiency of the cellular clearance processes promote intercellular transfer as an alternative pathway to remove toxic components?

Another issue is the interplay between exosomes and TNTs to harness synergistic effect on intercellular communication. In this context, the work of Mentor and Fisher is timely and highly relevant. The study illustrates, using high-resolution scanning electron microscopy (HR-SEM), that membrane-bound exosomes attached to the plasma membrane of brain endothelial cells (BEC), participate in TNT formation between the cells, and facilitate *in vitro* blood brain barrier (BBB) genesis. Research has also shown that exosomes released by BECs transfer specific cargo to recipient brain cells (Banks et al., 2020). BECs play pivotal roles in the permeability of the BBB, which is implicated in the progression of neurodegenerative pathologies. The study of Mentor and Fisher suggested TNT-mediated trans-permeability between BEC cells. Recently another study has shown that thrombospondin-1-containing exosomes released from breast cancer cells promote biogenesis of TNTs (Mahadik and Patwardhan, 2023). Furthermore, exosomes utilize TNTs as vehicles for efficient long-distance transfer of selective cargo to the recipient cells (Thayanithy et al., 2014). This remarkable synergy and the convergence between exosomes and TNTs presents a novel mechanism in the field of intercellular transfer (Figure 1).

**Figure 1:**
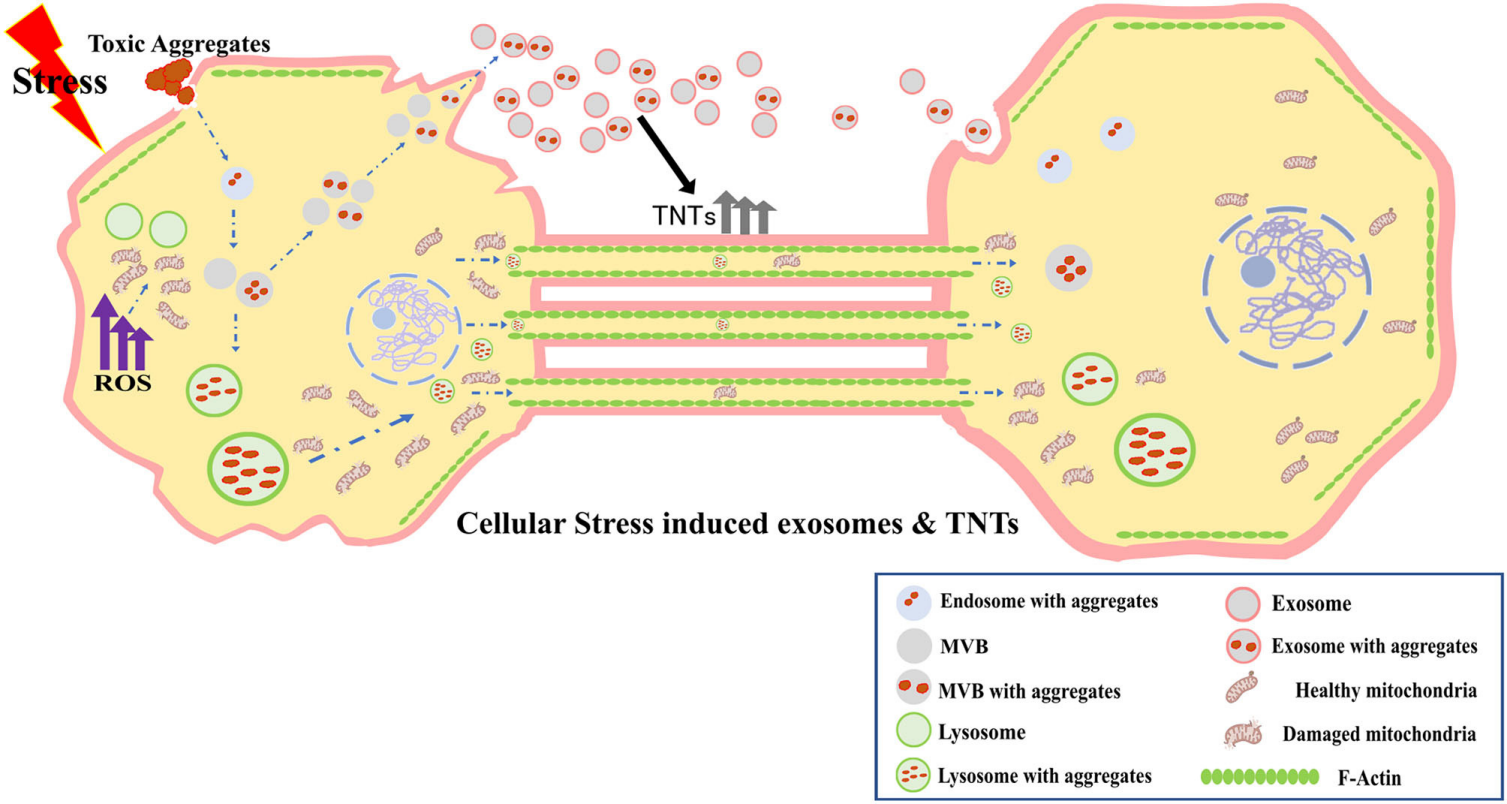
Cellular stress induced exosomes and TNTs, and their synergistic interactions in long-range intercellular transfer.

In conclusion, the synergistic interplay between exosomes and TNTs in long-range intercellular transfer is rapidly evolving as a promising area of cellular communication. Several important questions have been raised by recent advancements in this emerging field of research. What molecular mechanisms govern their on-demand biogenesis in response to cellular toxicities to rescue pathologies? How are exosomes selectively incorporated into TNTs? Do specific pathophysiological conditions enhance or restrict biogenesis of these intercellular conduits and their functionality? Addressing these questions could provide valuable insights into intercellular communications and their complexities, and will open up new avenues for therapeutic intervention.

## Author contributions

All authors listed have made a substantial, direct, and intellectual contribution to the work and approved it for publication.
